# The genome and proteome of a *Campylobacter coli *bacteriophage vB_CcoM-IBB_35 reveal unusual features

**DOI:** 10.1186/1743-422X-9-35

**Published:** 2012-01-27

**Authors:** Carla M Carvalho, Andrew M Kropinski, Erika J Lingohr, Sílvio B Santos, Jonathan King, Joana Azeredo

**Affiliations:** 1 IBB--Institute for Biotechnology and Bioengineering, Centre of Biological Engineering, Universidade do Minho, Campus de Gualtar, 4700-057 Braga, Portugal; 2 Laboratory for Foodborne Zoonoses, Public Health Agency of Canada, 110 Stone Road West, Guelph, ON N1G 3W4, Canada; 3 Department of Biology, Massachusetts Institute of Technology (MIT), Cambridge, MA, USA

**Keywords:** Bacteriophage, Genome, *Campylobacter*

## Abstract

**Background:**

*Campylobacter *is the leading cause of foodborne diseases worldwide. Bacteriophages (phages) are naturally occurring predators of bacteria, ubiquitous in the environment, with high host specificity and thus considered an appealing option to control bacterial pathogens. Nevertheless for an effective use of phages as antimicrobial agents, it is important to understand phage biology which renders crucial the analysis of phage genomes and proteomes. The lack of sequence data from *Campylobacter *phages adds further importance to these studies.

**Methods:**

vB_CcoM-IBB_35 is a broad lytic spectrum *Myoviridae Campylobacter *phage with high potential for therapeutic use. The genome of this phage was obtained by pyrosequencing and the sequence data was further analyzed. The proteomic analysis was performed by SDS-PAGE and Mass spectrometry.

**Results and conclusions:**

The DNA sequence data of vB_CcoM-IBB_35 consists of five contigs for a total of 172,065 bp with an average GC content of 27%. Attempts to close the gaps between contigs were unsuccessful since the DNA preparations appear to contain substances that inhibited Taq and ϕ29 polymerases. From the 210 identified ORFs, around 60% represent proteins that were not functionally assigned. Homology exists with members of the *Teequatrovirinae *namely for T4 proteins involved in morphogenesis, nucleotide metabolism, transcription, DNA replication and recombination. Tandem mass spectrometric analysis revealed 38 structural proteins as part of the mature phage particle.

**Conclusions:**

Genes encoding proteins involved in the carbohydrate metabolism along with several incidences of gene duplications, split genes with inteins and introns have been rarely found in other phage genomes yet are found in this phage. We identified the genes encoding for tail fibres and for the lytic cassette, this later, expressing enzymes for bacterial capsular polysaccharides (CPS) degradation, which has not been reported before for *Campylobacter *phages.

## Background

Recent publications indicate that *Campylobacter *is the leading cause of foodborne diseases worldwide, clearly surpassing other foodborne pathogens such as *Salmonella*. Measures commonly used to control foodborne pathogens have had little success against *Campylobacter*, which is a reflection of differences in the physiology, epidemiology and ecology of these organisms.

The renewed interest in phages as therapeutic agents has contributed to the rapid increase in the number of phages sequences described in the literature [1]. However, as far as *Campylobacter *phages are concerned, only two lytic phage genomes have been described so far: CP220, CPt10 [2]. The lack of sequence data from *Campylobacter *phages is probably due to the fastidious nature of their host bacterium which renders phage isolation tricky, and due to the refractory nature of their DNA leading to difficulties in genome characterization by common methods such as restriction fragment length polymorphism.

We recently reported the isolation, characterization and *in vivo *performance of the broad lytic spectrum *Campylobacter coli *phage vB_CcoM-IBB_35 (previously named phiCcoIBB35) which exhibited high potential for therapeutic use [3,4]. In fact, in a previous study this phage proved to be efficient in reducing the numbers of *C. coli *and *Campylobacter jejuni *by approximately 2 log 10 cfu/g in infected poultry [4]. This phage belongs to the *Myoviridae *family as do the majority of *Campylobacter *phages described in the literature [5,6] and has a genome size estimated to be 204 kbp by pulsed-field gel electrophoresis [4]. The majority of proteins encoded by phages with large genomes has no matches in the current sequence databases and has undiscovered functions [7]. Nevertheless, they have a conserved core of genes mainly involved in morphogenesis and in DNA and nucleotide processing [8].

We describe herein the genomic sequence and the proteomic analysis of *C. coli *phage vB_CcoM-IBB_35 (IBB_35) that exhibits homologies to T4-like phages.

## Methods

### Bacterial strains and phages

Phage IBB_35 belongs to the Centre for Biological Engineering **- **Institute for Biotechnology and Bioengineering private collection of phages (CEB-IBB, Minho University) and was isolated as part of the European Project "PhageVet-P". This phage was isolated from poultry intestinal contents and presents a broad lytic spectrum against food and clinical *C. coli *and *C. jejuni *strains. A wild type *C. coli *strain A11 isolated from poultry was used as the propagating strain for this phage [3,4].

### Phage purification

The phage lysate was precipitated using polyethylene glycol (PEG) 8,000 according to the procedure described by Sambrook and Russell [9] followed by purification through cesium chloride (CsCl) equilibrium gradient centrifugation. Briefly, the phage suspension was added to the top of five CsCl solutions with different densities (1.25, 1.3, 1.4, 1.5, 1.6) which were previously layered by increasing density under one another in a Beckman Ultraclear centrifuge tube. The gradient was centrifuged at 141,000 × g (28,000 rpm, Beckmann SW28 rotor) at 4°C for 2 h, and the band with highest opalescence was collected. A Millipore Centricon 20 spin filter was used to reduce the volume of recovered CsCl purified phage concentrate. The centrifuge was initially set to 6,500 × g at 4°C for 5 min, but then the spin times were adjusted as necessary in order to allow most of the liquid to pass through the filter. After the volume had been reduced, the concentrate was dialyzed in a 10 K Slide-A-Lyzer cassette (Pierce Biotechnology, Rockford, IL) against a first buffer (50 mM Tris, 100 mM MgSO_4_, 3 M NaCl; pH 7.5) for 1 h. Thereafter, the suspension was dialysed overnight against a second buffer (50 mM Tris, 100 mM MgSO_4_, 1 M NaCl; pH 7.5) followed by 1 h against a third buffer (50 mM Tris, 100 mM MgSO_4_, 100 mM NaCl; pH 7.5). After the third wash, the small volume was taken with a pipette and stored at 4°C.

### DNA extraction and purification

Phage DNA was extracted using the SDS-proteinase K protocol of Sambrook and Russell [9], precipitated with ethanol and resuspended in ultrapure water. An alternative methodology adapted from Moreira [10] was used in order to purify the phage sample for the PCR amplification. Briefly the phage sample was embedded in low melting point (LMP) agarose blocks and then immersed in a lysis buffer, followed by several washing steps. The final agarose plugs were cut in small pieces and used for the PCR reaction.

### Genome sequencing and analysis

DNA was submitted to the McGill University and Génome Québec Innovation Centre (Montréal, QC, Canada) for pyrosequencing, resulting in five large contigs (> 1,000 bp).

The genome was annotated using Kodon (Applied Maths, Austin, TX) and a variety of online tools http://molbiol-tools.ca were used at their default setting for genome and protein analysis. These included: tRNAScan-SE [11] for searching tRNA-encoding genes; TMHMM http://www.cbs.dtu.dk/services/TMHMM for prediction of transmembrane domains; Phobius [12] and SignalP [13] for prediction of signal peptides; BLASTP for screening for homology; DNAMan (Lynnon Corporation, Pointe-Claire, QC, Canada) for codon usage determination; and EditSeq (DNASTAR Inc, Madison, WI) for calculation of protein molecular weights (MW).

Promoters were screened using Kodon for the consensus sequence [-35]TTGACA_N15-17_TATAAT[-10] allowing for two mismatches. Potential rho-independent terminators were identified using MFOLD [14], after visually scanning for polyT tracts.

Comparisons between the genome of IBB_35 and other selected phage genomes were made at the nucleotide and at the proteomic level using Mauve [15] and CoreGenes [16], respectively, at their default settings.

### Proteomic analysis

Phage purified sample was resuspended in gel loading buffer [18.8 ml 1 M Tris pH 6.8, 6 g SDS (final concentration 2%), 15 ml 2-mercaptoehtanol, 30 ml glycerol, a small amount of bromophenol blue, qs 100 ml H_2_0] and denatured in a boiling water bath for 5 min. Proteins were separated by denaturing gel electrophoresis (SDS-polyacrylamide gel electrophoresis) on a one dimensional 12% gel. The marker used was the Precision Plus Protein, Unstained Standards (Bio-Rad Laboratories, Richmond, CA). The gel was stained using silver stain according to standard protocols [17]. The bands obtained were digested with trypsin and the peptides obtained were subsequently identified using electrospray ionization-tandem mass spectrometry (ESI-MS/MS). The MS (Mass spectrometry) data was analyzed using Scaffold [18].

### Nucleotide sequence accession number

In agreement with Kropinski *et al. *[19] that suggested the creation of a systemized nomenclature for phages, the *C. coli *phage was renamed accordingly, before being deposited in the GenBank. Therefore it was designed as vB_CcoM-IBB_35 representing: (vB) bacterial virus; (Cco) host *C. coli*; (M) the virus family *Myoviridae*, and (IBB_35) the common laboratory name.

The genome sequence of this phage was deposited in GenBank under the accession numbers: Contig1 [Genbank:HM246720], Contig 2 [Genbank:HM246721], Contig 3 [Genbank:HM246722], Contig 4 [Genbank:HM246723], and Contig 5 [Genbank:HM246724].

## Results and discussion

### Virological and genomic features of phage vB_CcoM-IBB_35

Phage IBB_35 is a member of the *Myoviridae *presenting an icosahedral head (average diameter of 100 nm) and a contractile tail (140 × 17 nm average length) with tail fibres at the distal end [3].

DNA sequencing of phage IBB_35 resulted into five large contigs: contig 1 (53,237 bp); contig 2 (51,534 bp); contig 3 (27,985 bp); contig 4 (14,701 bp) and contig 5 (24,608 bp) for a total of 172,065 bp. This value was smaller than the one estimated by pulsed-field gel electrophoresis (PFGE), i.e. 204 kbp.

**Figure 1:**
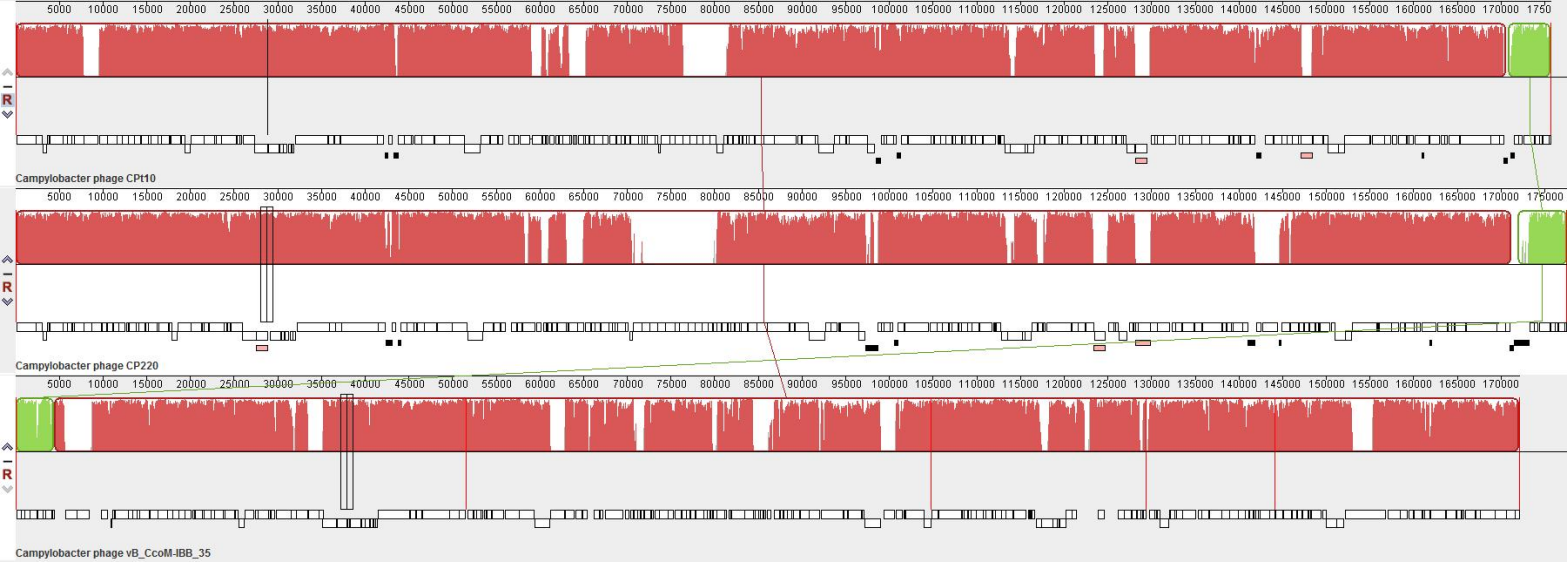
**Mauve progressive DNA alignment between vB_CcoM-IBB_35, CP220, CPt10**. The red bars represent the regions of homology and the white bars represent the regions with no homology (adapted from Mauve output) [15].

The five contigs obtained from the sequence of phage IBB_35 were aligned, using Mauve (Figure 1), with *Campylobacter *phages CP220 and CPt10 deposited in GenBank with accession numbers FN667788 and FN667789, respectively. Due to the high degree of sequence similarity between IBB_35 and the other two phages it was possible to align the 5 contigs accordingly: contig 2, contig 1, contig 5, contig 4, contig 3 with theoretical gaps of 100, 200, 300 and 400 bp. These results suggest that the genome is actually 173 kb. The discrepancy between the size derived from sequencing and that estimated by PFGE would suggest that this virus possesses extremely long terminal repeats or that the PFGE size was overestimated by almost 18%. Despite the fact that PFGE was performed by an ISO 17025 certified laboratory, this value is unlikely to correspond to the real value of IBB_35 genome size. This can be probably attributed to the fact that phage DNA appears to be strongly associated with a protein that not only interfered with PCR amplification leading to the failure of all attempts to bridge the small gaps, but also with the overestimation of the phage genome mass by PFGE. This phage is also insensitive to digestion by endonucleases suggesting that the DNA is, in some way, modified. These observations are totally at variance with genomic DNA from the host.

While we did not achieve complete genome closure we will discuss what we have found on each contig. When appropriate, some average values from each contig were calculated. The genes were named as "n-x" in which "n" represents the contig in which the gene is located (from 1 to 5) and "x" represents a number attributed to that gene and assigned in increasing order. However, after the alignment with phages CP220 and Cp10, this numbering system was altered since each of IBB_35 contigs, except contig 1, was inverted relative to the comparator genomes.

### ORFs and tRNA genes

IBB_35 has double-stranded DNA genome with an overall GC content of 27%, which is less than that of the host bacterium, *Campylobacter *spp (approximately 31%) [2].

**Figure 2:**
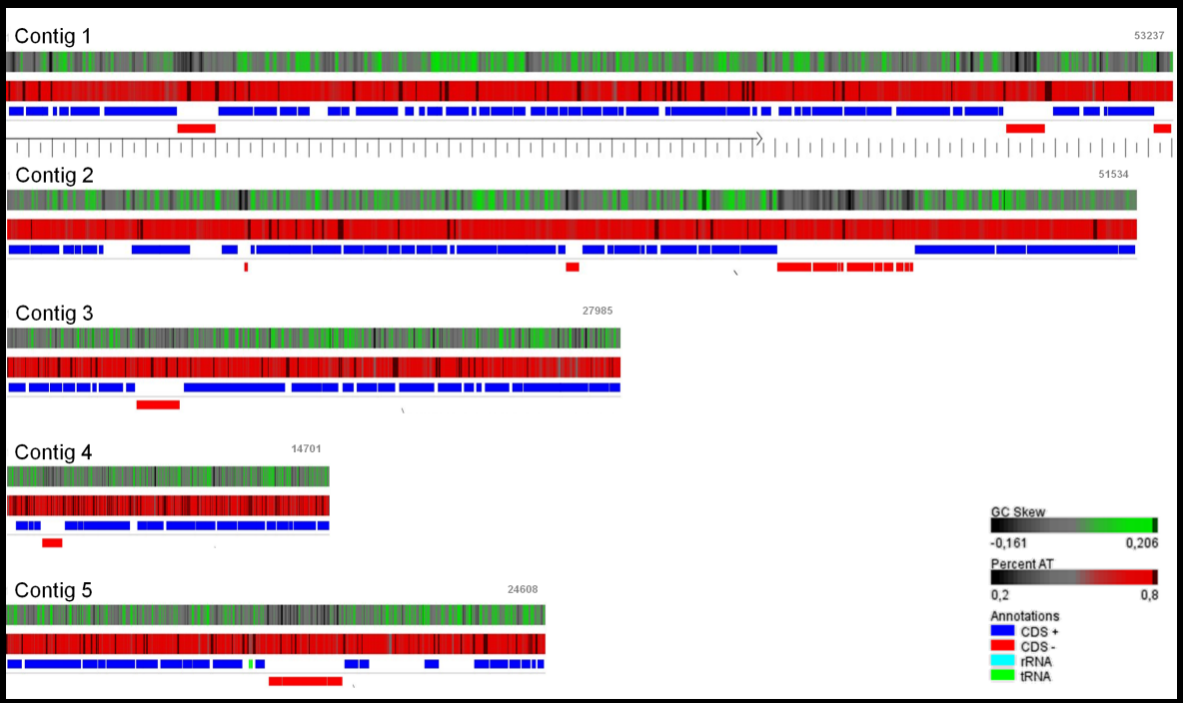
**Genetic and physical map of vB_CcoM-IBB_35 representing the 5 DNA contigs: (A) contig 2; (B) contig 1; (C) contig 5; (D) contig 4; (E) contig 3 (adapted from the GeneWiz output) **[21].

In the phage genome, 210 open reading frames (ORFs) were identified: 68 ORFs in contig 1; 62 ORFs in contig 2; 27 ORFs in contig 3; 22 ORFs in contig 4; and 31 ORFs in contig 5 (Figure 2). The majority of the ORFs (68%) were transcribed from the top strand, as it is described for many phages [20]. The overall percentage of coding sequence was 90%. Eighty four ORFs (40%) presented obvious similarity to proteins of known function and thus were tentatively assigned. An additional 109 (52%) gene products were found to resemble functionally unassigned proteins (Additional file 1: Table S1).

Phage IBB_35 was found to use ATG as the principle initiation codon (78.2%) which is in accord with the overall bacterial genomes deposited in the NCBI (National Center for Biotechnology Information) [22]. Other initiation codons were found in lower percentage: ATA in 8.2%, TTG in 6.1% and GTG in 4.9%. The rare initiation codons ATT (4.2%), ATC (3.1%) and CTG (2.6%) were also found in the IBB_35 genome.

In contig 5, two tRNA genes, Tyr-tRNA (GTA) and Arg-tRNA (TCT), were found close to each other, and between gene 5-16 and gene 5-17. Two rho-independent transcription terminators were identified in contig 1, contiguous to each other and after gene 1-60. The analysis of phage sequence revealed 22 putative promoters (Additional file 1: Table S1). Interestingly most of the promoters are present as duplicates before protein-encoding genes.

### Genome organization

The sequence data from IBB_35 reveal structural proteins and numerous genes involved in nucleotide metabolism, replication, morphogenesis, recombination and transcription with homology to T4 phages [23]. Therefore, the sequenced *Campylobacter *phage will be discussed as a T4-like phage and the genes from T4 phage will be designated, as in the literature, "gp" followed by a representative number.

#### Nucleotide metabolism

Phage IBB_35 contains numerous genes involved in nucleotide metabolism for which homologous proteins can be found in coliphage T4 [23]. The former comprise aerobic and anaerobic ribonucleotide-diphosphate reductase genes (gene 5-7 and gene 3-20; gene 5-3), deoxyuridine 5'-triphosphate nucleotidohydrolase (gene 2-44), thymidylate synthase (gene 3-22), thymidine kinase (gene 4-0), GTP cyclohydrolase (gene 5-18) and PhoH (gene 1-16). Therefore it can be assumed that phage IBB_35 has a pool of enzymes needed to accomplish the *de novo *nucleotide synthesis in aerobic and anaerobic environments.

The enzyme ribonucleotide-diphosphate reductase plays a central role in the *de novo *synthesis of deoxyribonucleoside triphosphates, which in turn are channeled into phage DNA replication. In fact, this enzyme generates deoxyribonucleotides through the reduction of the corresponding ribonucleotides [24-26]. The gene 5-7 and the gene 3-20 encode, respectively, the subunit α and subunit β of an aerobic ribonucleotide-diphosphate reductase, which is homologous to the protein encoded by genes *nrdA *and *nrdB *in phage T4 [23]. This enzyme is likely to be the limiting factor in the initiation and rate of deoxyribonucleotide synthesis in infected cells. In turn, the onset of phage DNA replication, which occurs soon after infection, is dependent on the turning-on of deoxyribonucleotide synthesis [24-26]. The presence of an anaerobic ribonucleotide-diphosphate reductase (gene 5-3) in IBB_35 is likely to enhance their efficiency in conditions that are very likely to occur since this phage infects *Campylobacter*, a microaerobic host [27,28].

Gene 2-44 encodes deoxyuridine 5'-triphosphate nucleotidohydrolase which is important for regulating the intracellular pool of dUTP since it catalyses the conversion of dUTP to dUMP. Consequently, since dUMP is the precursor for dTTP synthesis it provides an exclusive source of dUMP for *de novo *dTTP biosynthesis. The conversion of dUMP to dTMP is catalyzed by the enzyme thymidylate synthase [23,29] which is also encoded by a gene (gene 3-22) from this phage. Thymidine kinase is encoded by gene 4-0 and is thought to be a *salvage *enzyme since no thymidine is made biosynthetically but can only be made from the breakdown of dTMP [30].

#### Genome replication and recombination

Phage IBB_35, as a putative T4-like phage, is likely to use at different times in its life cycle, two major replication initiation mechanisms: the origin-dependent replication and the recombination-dependent replication [31]. In fact, *C. coli *phage IBB_35 seems to code for most components of its own replication complexes and for many enzymes that synthesize precursors for, or modify, DNA. It encodes all genes that constitute T4 phage replisome complex: a primosome composed of a primase/helicase (gene 5-17) homologous to T4 gp41, a primase (gene 2-42) homologous to gp61, and a primase homologous to gp59; a leading and a lagging strand holoenzyme composed of DNA polymerase (gene 5-29) homologous to gp43, sliding clamp-loader (gene 2-41, gene 2-27) homologous to gp44/gp62, and a sliding clamp protein (gene 5-20) homologous to gp45; and a single strand binding protein (gene 3-12) homologous to gp32 [23,32].

An interaction between the holoenzyme of IBB_35 and its primosome is likely to occur, as it happens in T4 [33]. In fact, helicases (gene 5-17) unwind dsDNA ahead of DNA polymerase (gene 5-29) and exposes ssDNA to which single-strand binding protein (gene 3-12) binds and thus prevents formation of DNA secondary structure and reannealing of the duplex.

The primase (gene 2-42) associates with the helicase (gene 5-17) and synthesizes short oligoribonucleotides that serve as the primers for the Okazaki fragments. The primers are later removed by RNase H (gene 2-40), the DNA polymerase fill the gaps so that DNA ligase (gene 2-18; homologous to gp30) can join the Okazaki fragments to form a continuous complementary strand [34,35]. It was also found in IBB_35 three genes encoding three topoisomerase II proteins with homology to T4 gp39 (gene 2-46) and gp52 (gene 4-10) and to a DNA gyrase (gene 3-13). These enzymes catalyze DNA interconversions and thus play an important role in replication, recombination and DNA repair [36].

The recombination-dependent replication at the 3' ends of D-loops is created by strand invasion, and is considered the predominant mode of replication late in infection [31]. In contig 4, some genes involved in this mechanism seems to define a cluster: DNA replication origin-binding helicase (gene 4-7); UvsW protein (gene 4-13) which has a helicase activity and leads to the inactivation of origin initiation [37,38]; gene 4-14 encoding an exonuclease (homologous to RecB exonuclease). The gene 1-15 encodes a protein homologous to gp2 of T4 phages, and is of extreme importance since it protects newly injected DNA from degradation by exonucleases [39,40].

#### Morphogenesis: Proteomic and *in silico* analysis

**Figure 3:**
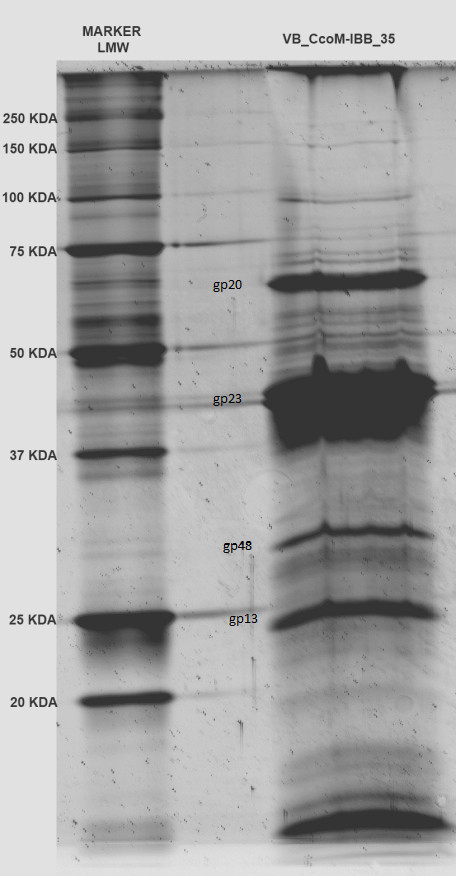
**SDS-PAGE of vB_CcoM-IBB_35 structural proteins (kDa)**.

The genes related to morphogenesis are distributed on each of the five contigs and do not define a cluster. Analysis of the data obtained from SDS-PAGE gel (Figure 3) and ESI-MS/MS allowed the identification of 38 predicted IBB_35 structural proteins with different sequence coverage percentage (Additional file 2: Table S2). From these proteins, 12 have homologues to the T4 structural proteins [23], namely tail tube proteins gp3 (gene 2-61) and gp19 (gene 1-14, gene 2-0, gene 3-14), tail sheath proteins gp18 (gene 1-7, gene 1-8, gene 2-15), major capsid head protein gp23 (gene 1-6), scaffold/prohead protease protein gp21 (gene 2-54), the portal vertex protein gp20 (gene 2-20), neck protein gp13 (gene 5-24), the baseplate wedge proteins gp6 (gene 2-5) and baseplate hub proteins gp48 (gene 3-1). In addition, a minor tail protein, a virion structural protein and 25 proteins with no homology were found in the data obtained by ESI-MS/MS (Additional file 2: Table S2). However, some genes present in the IBB_35 sequence that code for structural proteins homologous to T4 phage proteins were not detected by this method. Those identified by BLASTP as homologous include a baseplate hub gp51 (gene 2-49), an outer wedge baseplate subunit (gene 2-10) and two tail stabilizer proteins gp3 (gene 2-61) and gp15 (gene 3-15). The major percentage of sequence coverage of these predicted structural proteins was obtained for the major capsid protein (49%) which is in accord with other phages reported in the literature, and followed by a minor phage tail protein (38%).

Phage IBB_35 seems to have a lysozyme associated with the tail that would probably enhance DNA entrance into the host cell. This assumption is validated by the presence of the gene 1-33 in the phage sequence, which encodes a protein homologous to a baseplate subunit associated with a lysozyme. Moreover the gene 1-35 encodes a protein homologous to a T4 phage tail lysozyme.

The presence of three genes encoding the tail tube protein gp19 and the tail sheath protein gp18 is also in accordance with what has been reported for other *Campylobacter *phages [2].

The agreement between the predicted and observed protein molecular weights suggests that the majority of these proteins are not proteolytically modified. Thus, the ClpP protease encoded by gene 4-9 seems to have no activity on these proteins, but probably on others that were not identified during ESI-MS/MS, including the major prohead-scaffolding core protein gp22 (gene 1-5).

In phage IBB_35 some genes encoding functional proteins involved in the morphogenesis were identified. These include the gene encoding a chaperonin Cpn10 (gene 3-11) which is said to have ability to prevent or deter incorrect protein folding and aggregation [38] and the gene 3-8 encoding the RNA ligase 1 and tail fibre attachment catalyst, which promotes noncovalent joining of tail fibres to the phage baseplate. This last gene is, in fact, placed downstream the gene that encodes the tail fibres (gene 3-16), both participating in the last step of morphogenesis [32,41].

#### Rare features of phage IBB_35 genome

An interesting feature of phage IBB_35 is the fact that no evidence was found for the small subunit of the terminase complex which confers the specific DNA-binding/association properties and is usually found upstream of the large subunit in most of T4-like genomes [42,43]. Gene 2-52 clearly encodes the large subunit of terminase. Since we could not find the gene that encodes the small subunit of terminase, we are tempting to suggest that IBB_35 belongs to the rare group of phages that may only require the endonuclease and ATPase activity of the terminase large subunit in order to cleave and pack the DNA. Examples of these phages include: *Bacillus subtilis *phage ø29, *Erwinia *phage øEa21-4, coliphage rV5 and *Salmonella *phage Felix01 [44,45].

One of the unusual characteristics of phage IBB_35 is the high incidence of homing endonucleases and of split genes with inteins and introns. We observed that gene 2-52, encoding the large subunit of terminase, was interrupted by an intein and an intron that encloses a homing endonuclease (gene 2-51). This homing endonuclease (gene 2-51) has homology with HNH family endonuclease, *mobE*, which is usually found inserted between the large (*nrdA*) and small (*nrdB*) subunit genes of aerobic ribonucleotide reductase (RNR) of T-even phages T4, RB2, RB3, RB15, and LZ7 [46]. The coexistence of an intein and a intron in the same gene has, to our knowledge, been only reported for the *Bacillus subtilis *phage SPβ ribonucleotide reductase gene, and was considered an unlikely event to occur by chance [47]. The presence of an intron/intein and a homing endonuclease targeting the same gene normally results from a rare recombination event where the endonuclease is inserted into the intron/intein without affecting its splicing, thereby giving rise to a composite parasitic element that can move together between different hosts [48].

The gene encoding the PhoH protein (1-6) and the gene encoding the ribonucleotide-diphosphate reductase subunit alpha (5-7) are interrupted by two inteins. This phenomenon has never been observed before in these particular genes. Although no evidence has yet been forthcoming for a regulatory role for introns or inteins, and homing endonucleases they are likely to confer a selective advantage [49].

In phage IBB_35 sequence two genes (1-9 and 1-10) were found adjacent to each other and encoding the same protein which has homology to Hef (homing endonuclease-like function) [50]. These genes have been recently reported as existing between *nrdA *and *nrdB *(in place of the *mobE *gene in T4) in phage U5 [49] although in phage IBB_35 they are placed upstream of these two genes. Although Hef displays endonuclease activity it has no similarity to any known homing endonuclease. As it has cleavage sites close to its gene loci in the phage genomes it is suggested to be beneficial for the spreading of the homing endonuclease [49].

The genome of phage IBB_35 also contains six genes (1-41, 1-55, 2-28, 2-34, 4-1, 3-5) that encode proteins homologous to radical S-adenosyl-L-methionine (SAM) superfamily proteins. These proteins are very rare in phage genomes and to our knowledge have only been described for CP220 and CPt10 phages [2]. Nevertheless they are highly common in *Campylobacter *genomes (1,717 hits obtained using BLASTP). The high prevalence of genes encoding this protein, along with their wide distribution in the phage genome can be explained by the fact that these proteins catalyse diverse reactions such as isomerization, sulfur insertion, ring formation, anaerobic oxidation and protein radical formation. Moreover they function in DNA precursor, vitamin cofactor, antibiotic and in biodegradation pathways. In addition, these proteins can be useful to phage as they can be the activating enzymes for pyruvate formate-lysate and anaerobic ribonucleotide reductase [50,51]. The unusual methylations catalyzed by these enzymes can also protect the phage from the bacteria restriction enzymes which may explain the reason for the highly refractory nature of the DNA of IBB_35.

#### Host recognition

**Figure 4:**
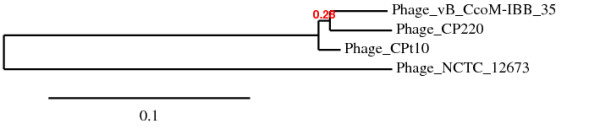
**Phylogenetic tree of the tail fibre gene based on the known sequences of *Campylobacter *phages: vB_CcoM-IBB35, CP220, Cpt10, NCTC 12673 (adapted from the Phylogeny.fr output) **[52].

The genes encoding for the tail fibres of phage IBB_35 were not immediately recognized by BLASTP. This might have been the explanation for Timms *et al. *[2] not report these genes in CP220 and Cpt10. However, gene 3-16 has homology with one of the genes encoding for the tail fibre proteins of *Campylobacter *phage NCTC 12673 (Szymanski, personal communication). Moreover, this gene is also homologous to proteins CBJ93981.1 and CBJ94379.1 from the phage CP220 and phage CPt10, respectively, described as encoding hypothetical proteins (Figure 4). Interestingly these two last proteins showed high sequence similarity (92.4%) whereas the protein product of 3-16 presented lower sequence similarity (48%) to CBJ94379.1 and to CBJ93981.1. Moreover gene 3-16 showed 66% of sequence similarity with the gene encoding the tail fibres of NCTC 12673. These dissimilarities can indicate different host specificities. It should be interesting to evaluate the lytic spectrum of each of these three phages against the same strains in order to assess if the small nucleotide differences among the putative gene encoding for tail fibre proteins is responsible for different host specificity.

#### Lysis

The two-part lysis system (lytic cassette) that is present in most dsDNA phages is composed of genes encoding a holin and cell wall hydrolases (generally called endolysins or lysins). At the end of the infection, the holin permeabilizes the cytoplasmic membrane allowing access of the phage lysin to its murein substrate [53-55].

In phage IBB_35, genomic analysis identified gene 5-22 as encoding a protein with high homology with a lytic murein transglycosylase and therefore likely to be the endolysin. This protein is similar to hypothetical proteins (CBJ93929.1, CBJ94327.1) of phage CP220 and CPt10, respectively.

The gene for the holin component is usually found directly upstream of the endolysin, sometimes even overlapping the latter [55]. However in IBB_35 the gene for the holin is likely to be gene 2-8, which is located distant from the lysin. Nevertheless the product of this gene contained a Phage_holin_3 family (Pfam PF05106.5) motif and two transmembrane domains, which are considered typical characteristics of holins [53,56]. This protein shows great than 90% sequence identity to hypothetical proteins (CBJ93848.1, CBJ94240.1) of phage CP220 and CPt10.

We also identified a number of ORFs that encode proteins potentially involved in the carbohydrate metabolism. Gene 3-23, encodes a polysaccharide deacetylase, and gene 3-24, encodes a LmbE-like protein. Both of these proteins have been shown to be involved in the degradation of polysaccharides in bacteria [57,58]. We hypothesized that the proteins encoded by these genes are probably involved in the degradation of the bacteria surface polysaccharides to enhance progeny release or infection [58,59].

In phage IBB_35, gene 2-32 encodes a UDP-glucose dehydrogenase. In many bacterial strains such as *Campylobacter *species, UDP-glucose dehydrogenase catalyzes the NAD^+^-dependent oxidation of UDP-glucose to UDP-glucuronic acid which is necessary for the synthesis of capsular polysaccharide (CPS). It was recently reported that the over-expression of this enzyme inhibits the formation of the K5 capsular polysaccharide in *E. coli *[60]. If a similar mechanism exists in *Campylobacter *then gene 2-32 could be associated with a regulation mechanism that inhibits CPS synthesis enhancing phage burst. Homologs exist to hypothetical proteins (CBJ93828.1, CBJ94221.1) of phage CP220 and CPt10, respectively. Moreover these proteins showed high degree of sequence similarity (more than 90%).

## Conclusions

Analysis of the genome and proteome of phage IBB_35 reinforces the observation that *Myoviridae *group II *Campylobacter *phages are closely related, and display a distant relationship to the T4-like phages. However, they do contain some features never or rarely found in T4-like phages: radical SAM, presence of both inteins and introns in single genes and enzymes involved in carbohydrate metabolism. We were able to identify, for the first time, the lytic enzyme duo of *Campylobacter *phages, which encodes the endolysin (lysozyme), and holin together with potential CPS degrading enzymes. We also identified a gene likely to encode a tail fibre protein, whose functional analysis could contribute to development of a tool to specifically and physically enrich for *Campylobacter*.

## Competing interests

The authors declare that they have no competing interests.

## Authors' contributions

CMC was responsible for samples preparation, genome annotation and analysis, SDS-PAGE experiments, and drafted the manuscript. EJL participated in the phage DNA extraction and purification and in the PCR experiments. AMK participated in the design of the study, assisted in the sequencing annotation and alignment and made substantial contributions to the sequencing data analysis. SS assisted in drafting the manuscript and participated in the genome analysis by the use of online tools. JK provided the expertise in the protein extraction, purification and *in silico *analysis. JA conceived the study, and participated in its design and coordination and helped to draft the manuscript. All authors read and approved the final manuscript.

## Supplementary Material

Additional file 1 **Table S1 **Identification of IBB_35 predicted structural proteins. Click here for file

Additional file 2 **Table S2 **Phage IBB_35 genome annotation. Click here for file
