# Supplementary material for: The genome and proteome of a Campylobacter coli bacteriophage vB_CcoM-IBB_35 reveal unusual features
Source: Virol J. 2012 Jan 27;9:35. doi: 10.1186/1743-422X-9-35 (PMC3322345; doi:10.1186/1743-422X-9-35)
Supplement: Additional file 1 — Table S1 Identification of IBB_35 predicted structural proteins. [file 1743-422X-9-35-S1.DOC]

Table S1 – Phage IBB_35 genome annotation

| **CONTIG 2** | | | | | | | |
| --- | --- | --- | --- | --- | --- | --- | --- |
| **ORFs** | **Strand** | **Start** | **End** | **AA Length** | **Product** | **Note (Sequence similarity to):** | **MW** |
| **P2-61** | + | 14 | 40 | 27 |  |  |  |
| **gene 2-61** | + | 95 | 598 | 504 | tail completion and sheath stabilizer protein | gp3 tail completion and sheath stabilizer protein[Enterobacteria phage Phi1] | 18,803 |
| **gene 2-60** | + | 608 | 1042 | 435 | hypothetical protein | No homologs | 16,224 |
| **gene 2-59** | + | 1064 | 1600 | 537 | putative protein | No homologs or RBS | 20,583 |
| **gene 2-58** | + | 1572 | 2399 | 828 | putative protein | No homologs or RBS | 31,554 |
| **gene 2-57** | + | 2560 | 3072 | 513 | conserved hypothetical protein | NP_860298.1 hypothetical protein HH0767  [Helicobacter hepaticus ATCC 51449] | 18,912 |
| **gene 2-56** | + | 2941 | 3072 | 132 | conserved hypothetical protein | ZP_00372096.1 hypothetical protein CUP0925 [Campylobacter upsaliensis RM3195] | 4,611 |
| **gene 2-55** | + | 3081 | 3395 | 315 | putative threonine dehydratase | ZP_02143498.1 threonine dehydratase [Roseobacter litoralis Och 149] | 11,908 |
| **gene 2-54** | + | 3448 | 4122 | 675 | prohead core scaffold and protease | NP_899607.1 gp21 [Vibrio phage KVP40] | 24,904 |
| **P 2-53** | + | 4152 | 4178 | 27 |  |  |  |
| **gene 2-53** | + | 4193 | 4399 | 207 | hypothetical protein | No homologs | 7,795 |
| **gene 2-52** | + | 4502 | 4923 | 422 | terminase, large subunit exon 1 |  | 27,424 |
| **Intein** | + | 4924 | 5374 | 451 |  |  |  |
| **gene 2-52** | + | 5375 | 5623 | 249 | terminase, large subunit exon 2 |  |  |
| **Intron** | + | 5624 | 8653 | 3030 |  |  |  |
| **gene 2-51** | + | 5709 | 6974 | 1266 | conserved hypothetical protein | YP_215339.1 hypothetical protein SC0352 [Salmonella enterica subsp. enterica serovar Choleraesuis str. SC-B67] | 49,703 |
| **gene 2-50** | + | 6971 | 8362 | 1392 | MobE homing endonuclease | NP_049844.1 MobE homing endonuclease [Enterobacteria phage T4] | 54,276 |
| **gene 2-52** | + | 8654 | 9775 | 1122 | terminase, large subunit exon 3 |  |  |
| **gene 2-49** | + | 9791 | 10531 | 741 | base plate protein | YP_656406.1 gp51 base plate protein [Aeromonas phage 25] | 28,627 |
| **gene 2-48** | - | 10841 | 10996 | 156 | hypothetical protein | No homologs | 5,576 |
| **P2-47** | + | 11031 | 11059 | 29 |  |  |  |
| **P2-47'** | + | 11059 | 11086 | 28 |  |  |  |
| **gene 2-47** | + | 11110 | 11298 | 189 | hypothetical protein | No homologs | 7,537 |
| **gene 2-46** | + | 11378 | 12889 | 1512 | topoisomerase II | NP_049621.1 gp39 topoisomerase II, large subunit, N-terminal region [Enterobacteria phage T4] | 57,379 |
| **gene 2-45** | + | 12889 | 13413 | 525 | conserved hypothetical protein | YP_001224812.1 hypothetical protein SynWH7803_1089 [Synechococcus sp. WH 7803] | 20,093 |
| **gene 2-44** | + | 13423 | 13881 | 459 | deoxyuridine 5-triphosphate nucleotidohydrolase | YP_002154348.1 deoxyuridine 5'-triphosphate nucleotidohydrolase, DUTP [Bacillus phage IEBH] | 16,947 |
| **gene 2-43** | + | 13923 | 14165 | 243 | hypothetical protein | No homologs | 9,347 |
| **gene 2-42** | + | 14178 | 15257 | 1080 | primase | NP_899268.1 gp61 [Vibrio phage KVP40] | 41,211 |
| **gene 2-41** | + | 15355 | 16269 | 915 | sliding clamp loader | YP_195157.1 sliding clamp loader gp44 [Synechococcus phage S-PM2] | 34,578 |
| **gene 2-40** | + | 16280 | 17338 | 1059 | RNase H | NP_891817.1 RnaseH ribonuclease [Enterobacteria phage RB49] | 41,640 |
| **gene 2-39** | + | 17397 | 17945 | 549 | conserved Hypothetical protein | NP_001048585.1 Os02g0826200 [Oryza sativa (japonica cultivar-group)] | 20,833 |
| **gene 2-38** | + | 17990 | 18622 | 633 | minor tail protein | NP_042314.1 minor tail protein [Lactococcus phage bIL67] | 23,617 |
| **gene 2-37** | + | 18291 | 19368 | 1078 | hypothetical protein | No homologs | 26,369 |
| **gene 2-36** | + | 19383 | 20081 | 699 | hypothetical protein | No homologs | 26,976 |
| **gene 2-35** | + | 20216 | 20422 | 207 | hypothetical protein | No homologs | 8,104 |
| **gene 2-34** | + | 20523 | 21317 | 795 | putative Radical SAM | YP_001490946.1 radical SAM domain-containing protein [Arcobacter butzleri RM4018] | 30,286 |
| **gene 2-33** | + | 21317 | 21577 | 261 | hypothetical protein | No homologs | 9,636 |
| **gene 2-32** | + | 21587 | 22360 | 774 | putative UDP-glucose dehydrogenase | YP_016298.1 UDP-glucose dehydrogenase [Mycoplasma mobile 163K] | 29,334 |
| **gene 2-31** | + | 22385 | 22786 | 402 | hypothetical protein | No homologs | 14,810 |
| **gene 2-30** | + | 22786 | 23724 | 939 | hypothetical protein | No homologs | 36,566 |
| **gene 2-29** | + | 23702 | 23923 | 222 | hypothetical protein | No homologs | 8,223 |
| **gene 2-28** | + | 23925 | 25028 | 1104 | putative Radical SAM | ZP_01371679.1 Radical SAM [Desulfitobacterium hafniense DCB-2] | 42,658 |
| **P2-27** | + | 25079 | 25105 | 27 |  |  |  |
| **gene 2-27** | + | 25139 | 25471 | 333 | clamp-loader subunit | YP_239017.1 gp62 clamp-loader subunit [Enterobacteria phage RB43] | 12,728 |
| **gene 2-26** | - | 25499 | 26104 | 606 | hypothetical protein | No homologs | 21,911 |
| **gene 2-25** | + | 26246 | 27265 | 1020 | hypothetical protein | No homologs or RBS | 39,734 |
| **gene 2-24** | + | 27387 | 27680 | 294 | hypothetical protein | No homologs | 10,994 |
| **gene 2-23** | + | 27707 | 28894 | 1188 | virion structural protein | YP_001429654.1 virion structural protein [Bacillus phage 0305phi8-36] | 44,049 |
| **gene 2-22** | + | 28905 | 29108 | 204 | hypothetical protein | No homologs | 7,875 |
| **gene 2-21** | + | 29176 | 29661 | 486 | EndoVII packaging and recombination endonuclease | NP_891632.1 endoVII packaging and recombination endonuclease VII [Enterobacteria phage RB49] | 18,523 |
| **gene 2-20** | + | 29823 | 31475 | 1653 | portal vertex protein of head | NP_861872.1 gp20 portal vertex protein of head [Enterobacteria phage RB69] | 64,126 |
| **gene 2-19** | + | 31551 | 32102 | 552 | conserved hypothetical protein | hypothetical protein Tc00.1047053510073.75 [Trypanosoma cruzi strain CL Brener] | 20,824 |
| **gene 2-18** | + | 32154 | 33443 | 1290 | DNA ligase | YP_001469557.1 gp30 DNA ligase [Enterobacteria phage Phi1] | 49,254 |
| **gene 2-17** | + | 33453 | 34652 | 1200 | putative tryptophan halogenase | YP_497163.1 tryptophan halogenase [Novosphingobium aromaticivorans DSM 12444] | 46,392 |
| **gene 2-16** | + | 34649 | 35152 | 504 | hypothetical protein | No homologs | 19,961 |
| **gene 2-15** | - | 35141 | 36691 | 1551 | tail sheath protein | YP_239196.1 gp18 tail sheath monomer [Enterobacteria phage RB43] | 59,193 |
| **gene 2-14** | - | 36761 | 37882 | 1122 | transposase, IS605 OrfB family | YP_001666049.1 IS605 family transposase OrfB [Thermoanaerobacter pseudethanolicus ATCC 33223] | 42,778 |
| **gene 2-13** | - | 37900 | 38007 | 108 | hypothetical protein | No homologs | 4,197 |
| **gene 2-12** | - | 38033 | 38155 | 123 | putative transcriptional regulator | YP_173710.1 MarR family transcriptional regulator [Bacillus clausii KSM-K16] | 4,809 |
| **gene 2-11** | - | 38324 | 39526 | 1203 | hypothetical protein | No homologs | 45,586 |
| **gene 2-10** | - | 39590 | 39952 | 363 | baseplate wedge subunit | NP_891745.1 baseplate wedge subunit [Enterobacteria phage RB49] | 13,834 |
| **gene 2-9** | - | 40005 | 40424 | 420 | hypothetical protein | No homologs | 14,472 |
| **gene 2-8** | - | 40571 | 40897 | 327 | hypothetical protein | No homologs | 11,981 |
| **gene 2-7** | - | 40958 | 41170 | 213 | conserved hypothetical protein | NP_860295.1 hypothetical protein HH0764 [Helicobacter hepaticus ATCC 51449] | 8,453 |
| **gene 2-6** | - | 41179 | 41331 | 153 | hypothetical protein | YP_214342.1 T4-like baseplate wedge [Prochlorococcus phage P-SSM2] | 5,659 |
| **gene 2-5** | + | 41422 | 45066 | 3645 | base plate wedge | YP_195114.1 baseplate wedge subunit gp6 [Synechococcus phage S-PM2] | 138,284 |
| **gene 2-4** | + | 45135 | 45803 | 669 | conserved hypothetical protein | XP_663924.1 hypothetical protein AN6320.2 [Aspergillus nidulans FGSC A4] | 25,471 |
| **gene 2-3** | + | 45814 | 46479 | 666 | hypothetical protein | no homologs | 25,781 |
| **gene 2-2** | + | 46536 | 49658 | 3123 | hypothetical protein | No homologs | 120,475 |
| **gene 2-1** | + | 49669 | 50697 | 1029 | putative protein | No homologs or RBS | 40,671 |
| **gene 2-0** | + | 50713 | 51471 | 759 | tail tube protein | NP_861871.1 gp19 tail tube protein [Enterobacteria phage RB69] | 28,691 |
|  |  |  |  |  |  |  |  |
| **CONTIG 1** | | | | | | | |
| **ORFs** | **Strand** | **Start** | **End** | **AA Length** | **Product** | **Note (Sequence similarity to):** | **MW** |
|  |  |  |  |  |  |  |  |
| **gene 1-0** | + | 160 | 819 | 660 | hypothetical protein | no homologs | 26,309 |
| **gene 1-1** | + | 917 | 1189 | 273 | hypothetical protein | no homolgs | 10,434 |
| **gene 1-2** | + | 1183 | 1683 | 501 | sigma factor involved in late transcription | YP_214376.1 T4-like sigma factor, late transcription [Prochlorococcus phage P-SSM2];no RBS | 19,476 |
| **gene 1-3** | + | 1680 | 1934 | 255 | hypothetical protein | no homologs | 10,055 |
| **gene 1-4** | + | 2140 | 2325 | 186 | hypothetical protein | no homologs | 6,958 |
| **gene 1-5** | + | 2438 | 2872 | 435 | major prohead-scaffolding core protein | NP_899608.1 gp22 [Vibrio phage KVP40] | 16,051 |
| **gene 1-6** | + | 2944 | 4278 | 1335 | major capsid protein | YP_214367.1 T4-like major capsid protein gp23 [Prochlorococcus phage P-SSM2] | 48,563 |
| **gene 1-7** | + | 4504 | 6069 | 1566 | tail sheath protein | YP_214361.1 T4-like tail sheath protein gp18 [Prochlorococcus phage P-SSM2] | 56,184 |
| **gene 1-8** | + | 6073 | 7809 | 1737 | gp18, tail sheath protein | NP_899602.1 tail sheath protein [Vibrio phage KVP40] | 64,208 |
| **gene 1-9** | - | 7844 | 9568 | 1725 | Hef | ABI48935.1Hef [Bacteriophage U5] | 67,919 |
| **gene 1-10** | + | 9688 | 11283 | 1596 | Hef | YP_874152.1 hypothetical protein YS40_139 [Thermus phage phiYS40], longest ORF, noobvious RBS | 62,987 |
| **gene 1-11** | + | 11328 | 12359 | 1032 | conserved hypothetical protein | NP_835697.1 probable poly A polymerase [Rhodothermus phage RM378] | 39,916 |
| **P1-12** | **+** | 12455 | 12482 |  |  |  |  |
| **gene 1-12** | + | 12505 | 13281 | 777 | hypothetital protein | no homologs | 29,745 |
| **gene 1-13** | + | 13323 | 13865 | 543 | hypothetical protein | no homologs | 20,535 |
| **gene 1-14** | + | 15299 | 15667 | 369 | tail tube protein | YP_195137.1 tail tube protein gp19 [Synechococcus phage S-PM2] | 21,708 |
| **gene 1-15** | + | 14677 | 15267 | 591 | DNA end protector protein | YP_195237.1 DNA end protector protein gp2 [Synechococcus phage S-PM2] ] | 14,419 |
| **P1-16** | + | 15871 | 15899 | 29 |  |  |  |
| **gene 1-16** | + | 15971 | 17890 | 1920 | PhoH family protein with Intein | YP_001489001.1 PhoH family protein [Arcobacter butzleri RM4018] | 72,777 |
|  | + | 16019 | 17113 | 1095 | Exon 1 - PhoH protein |  |  |
|  | + | 17114 | 17533 | 420 | Intein in PhoH CDS |  |  |
|  | + | 17534 | 17890 | 357 | Exon 2 - PhoH protein |  |  |
| **P1-17** | + | 17998 | 18024 | 27 |  |  |  |
| **P1-17'** | + | 18026 | 18052 | 27 |  |  |  |
| **gene 1-17** | + | 18214 | 18624 | 411 | hypothetical protein | No homologs | 16,427 |
| **P1-18** | + | 18752 | 18780 | 29 |  |  |  |
| **gene 1-18** | + | 18846 | 19124 | 279 | hypothetical protein | No homologs | 10,859 |
| **gene 1-19** | + | 18946 | 19068 | 123 | hypothetical protein | No homologs | 4,930 |
| **P1-20** | + | 19121 | 19149 | 29 |  |  |  |
| **P1-20'** | + | 19140 | 19168 | 29 |  |  |  |
| **gene 1-20** | + | 19222 | 19665 | 444 | conserved hypothetical protein | YP_001398190.1 phage protein [Campylobacter jejuni subsp. doylei 269.97] | 17,534 |
| **gene 1-21** | + | 19662 | 19931 | 270 | conserved hypothetical protein | ZP_00368024.1 response regulator [Campylobacter coli RM2228] | 10,620 |
| **gene 1-22** | + | 20073 | 20312 | 240 | hypothetical protein | no homologs | 9,244 |
| **gene 1-23** | + | 20309 | 20407 | 99 | hypothetical protein | no homologs | 3,979 |
| **gene 1-24** | + | 20404 | 21132 | 729 | hypothetical protein | no homologs | 28,373 |
| **gene 1-25** | + | 21240 | 21458 | 219 | hypothetical protein | no homologs | 8,480 |
| **gene 1-26** | + | 21593 | 22081 | 489 | hypothetical protein | no homolgs | 18,529 |
| **gene 1-27** | + | 22137 | 23123 | 987 | conserved hypothetical protein | no homolgs | 36,787 |
| **gene 1-28** | + | 23138 | 23701 | 564 | conserved hypothetical protein | YP_001490947.1 hypothetical protein Abu_2060 [Arcobacter butzleri RM4018] | 21,778 |
| **gene 1-29** | + | 23937 | 24605 | 669 | hypothetical protein | no homologs | 25,744 |
| **gene 1-30** | + | 24670 | 25212 | 543 | conserved hypothetical protein | ZP_00368743.1 membrane associated lipoprotein precursor [Campylobacter lari RM2100] | 21,402 |
| **gene 1-31** | + | 25250 | 25615 | 366 | hypothetical protein | no homologs | 13,716 |
| **gene 1-32** | + | 25655 | 26233 | 579 | conserved hypothetical protein | YP_700808.1 transketolase, N-terminal subunit [Rhodococcus sp. RHA1] | 22,166 |
| **gene 1-33** | + | 26296 | 27180 | 885 | putative Rhs element Vgr family protein | ZP_01907010.1 Rhs element Vgr family protein [Plesiocystis pacifica SIR-1]. | 32,737 |
| **gene 1-34** | + | 27220 | 27630 | 411 | hypothetical protein | no homologs | 15,273 |
| **gene 1-35** | + | 27630 | 27923 | 294 | conserved hypothetical protein | YP_656366.1 gp5.4 conserved hypothetical protein [Aeromonas phage 25] | 9,544 |
| **gene 1-36** | + | 27948 | 28199 | 252 | hypothetical protein | No homologs | 9,836 |
| **gene 1-37** | + | 28285 | 28722 | 438 | hypothetical protein | No homologs | 16,904 |
| **gene 1-38** | + | 28719 | 29591 | 873 | putative protein | No homologs | 33,908 |
| **gene 1-39** | + | 29610 | 29801 | 192 | hypothetical protein | no homologs | 7,723 |
| **gene 1-40** | + | 30078 | 30311 | 234 | hypothetical protein | no homologs | 8,607 |
| **gene 1-41** | + | 30349 | 31875 | 1527 | conserved hypothetical protein | YP_002016112.1 Radical SAM domain protein [Prosthecochloris aestuarii DSM 271] | 60,524 |
| **gene 1-42** | + | 31885 | 32856 | 972 | conserved hypothetical protein | YP_055346.1 glycine amidinotransferase [Propionibacterium acnes KPA171202] | 36,969 |
| **gene 1-43** | + | 32880 | 33050 | 171 | hypothetical protein | no homologs | 6,826 |
| **gene 1-44** | + | 33047 | 33529 | 483 | putative protein | No homologsno obvious RBS | 19,172 |
| **gene 1-45** | + | 33520 | 33675 | 156 | hypothetical protein | no homologs | 6,088 |
| **gene 1-46** | + | 33677 | 33940 | 264 | putative protein | No homologsno obvious RBS | 10,502 |
| **gene 1-47** | + | 34053 | 34283 | 231 | putative protein | No homologs no obvious RBS | 9,255 |
| **gene 1-48** | + | 34455 | 34910 | 456 | hypothetical protein | no homologs | 17,685 |
| **gene 1-49** | + | 35251 | 35838 | 588 | hypothetical protein | no homologs | 22,861 |
| **gene 1-50** | + | 35989 | 36285 | 297 | hypothetical protein | no homologs | 11,577 |
| **gene 1-51** | + | 36327 | 36719 | 393 | conserved hypothetical protein | YP_178588.1 putative lipoprotein [Campylobacter jejuni RM1221] | 14,517 |
| **gene 1-52** | + | 36779 | 37252 | 474 | hypothetical protein | no homologs | 18,642 |
| **gene 1-53** | + | 37267 | 37500 | 234 | hypothetical protein | No homolgs | 9,099 |
| **gene 1-54** | + | 37500 | 38177 | 678 | hypothetical protein | No homolgs | 27,043 |
| **gene 1-55** | + | 38262 | 39233 | 972 | conserved hypothetical protein | ZP_02636793.1 radical SAM domain protein [Clostridium perfringens B str. ATCC 3626] | 38,327 |
| **gene 1-56** | + | 39251 | 40417 | 1167 | hypothetical protein | no homologs | 44,446 |
| **gene 1-57** | + | 40619 | 43090 | 2472 | conserved hypothetical protein | NP_641412.1| phage-related tail protein [Xanthomonas axonopodis pv. citri str. 306] | 92,730 |
| **gene 1-58** | + | 43215 | 43640 | 426 | conserved hypothetical protein | XP_001914634.1 hypothetical protein EHI_093460 [Entamoeba histolytica HM-1:IMSS] | 15,983 |
| **gene 1-59** | + | 43747 | 45261 | 1515 | hypothetical protein | no homologs | 57,159 |
| **gene 1-60** | + | 45291 | 45515 | 225 | hypothetical protein | no homologs | 8,496 |
| **terminator** | + | 45538 | 45571 | 34 | Rho-independent terminator found using MFOLD |  |  |
| **terminator** | + | 45543 | 45578 | 36 | Rho-independent terminator found using MFOLD |  |  |
| **gene 1-61** | - | 45638 | 47392 | 1755 | hypothetical protein | No homologs | 64,565 |
| **P1-61** | - | 47653 | 47680 | 28 |  |  |  |
| **P1-61'** | + | 47774 | 48988 | 1215 |  |  |  |
| **gene 1-62** | + | 47774 | 48988 | 1215 | transposase | NP_632445.1 transposase [Methanosarcina mazei Go1] | 46,578 |
| **gene 1-63** | + | 49165 | 49917 | 753 | hypothetical protein | no homologs | 28,117 |
| **gene 1-64** | + | 50092 | 50253 | 162 | hypothetical protein | No homologs | 6,205 |
| **gene 1-65** | + | 50092 | 50253 | 162 | hypothetical protein | No homologs | 6,205 |
| **gene 1-66** | + | 50293 | 52380 | 2088 | conserved hypothetical protein | XP_453342.1 unnamed protein product [Kluyveromyces lactis] | 80,554 |
| **gene 1-67** | - | 52377 | 53159 | 783 | hypothetical protein | No homologs | 29,304 |
|  |  |  |  |  |  |  |  |
|  |  |  |  |  |  |  |  |
| **CONTIG 5** | | | | | | | |
| **ORFs** | **Strand** | **Start** | **End** | **AA Length** | **Product** | **Note (Sequence similarity to):** | **MW** |
| **gene 5-30** | + | 63 | 722 | 660 | putative transcriptional regulator | YP_001878480.1 Gene info exsB protein [Akkermansia muciniphila ATCC BAA-835] | 23,690 |
| **P5-29** | + | 727 | 755 |  |  |  |  |
| **gene 5-29** | + | 853 | 3426 | 2574 | DNA polymerase | YP_195168.1 Gene info DNA polymerase gp43 [Synechococcus phage S-PM2] | 99,273 |
| **gene 5-28** | + | 3500 | 3763 | 264 | hypothetical protein | No homologs | 10,352 |
| **gene 5-27** | + | 3763 | 4176 | 414 | putative protein | No homologs or RBS | 16,702 |
| **gene 5-26** | + | 4197 | 4541 | 345 | putative protein | No homologs or RBS | 13,971 |
| **gene 5-25** | + | 4584 | 5183 | 600 | hypothetical protein | No homologs | 23,245 |
| **gene 5-24** | + | 5183 | 5890 | 708 | neck protein | ABC95179.1 GP13-neck protein [Stenotrophomonas phage SMB14] | 26,874 |
| **gene 5-23** | + | 5943 | 6263 | 321 | hypothetical protein | No homologs | 12,554 |
| **gene 5-22** | + | 6253 | 6927 | 675 | hypothetical protein | No homologs | 26,251 |
| **gene 5-21** | + | 7047 | 7358 | 312 | hypothetical protein | No homologs | 11,637 |
| **gene 5-20** | + | 7355 | 8047 | 693 | sliding clamp protein | YP_195156.1 sliding clamp gp45 [Synechococcus phage S-PM2] | 26,408 |
| **gene 5-19** | + | 8072 | 8503 | 432 | neutrophil-activating protein A | YP_665108.1 neutrophil-activating protein A [Helicobacter acinonychis str. | 16,950 |
| **gene 5-18** | + | 8513 | 9292 | 780 | GTP cyclohydrolase | YP_677945.1 GTP cyclohydrolase I [Cytophaga hutchinsonii ATCC 33406] | 29,855 |
| **gene 5-17** | + | 9445 | 10782 | 1338 | primase/helicase | YP_214418.1 T4-like DNA primase-helicase [Prochlorococcus phage P-SSM2] | 50,776 |
| **P5-16** | + | 10794 | 10822 | 29 |  |  |  |
| **tRNA** | + | 11083 | 11159 | 77 | tRNA-Arg (tct) found using ARAGORN |  |  |
| **tRNA** | + | 11171 | 11256 | 86 | tRNA-Tyr_(gta ) found using ARAGORN |  |  |
| **gene 5-16** | + | 11383 | 11520 | 138 | hypothetical protein | No homologs | 5,363 |
| **gene 5-15** | + | 11513 | 11806 | 294 | putative protein | No homologs or RBS | 10,984 |
| **gene 5-14** | - | 11999 | 12592 | 594 | conserved hypothetical protein | ABU53782.1 cje0230 [Campylobacter phage CGC-2007] | 22,733 |
| **gene 5-13** | - | 12603 | 13859 | 1257 | hypothetical protein | No homologs, longest possible ORF, no observable RBS | 46,414 |
| **gene 5-12** | - | 13856 | 14371 | 516 | hypothetical protein | No homologs | 20,056 |
| **gene 5-11** | - | 14381 | 14644 | 264 | hypothetical protein | No homologs | 9,722 |
| **gene 5-10** | - | 14665 | 15354 | 690 | hypothetical protein | No homologs | 25,731 |
| **Promoter** | + | 15348 | 15376 | 29 |  |  |  |
| **gene 5-9** | + | 15445 | 16074 | 630 | hypothetical protein | No homologs | 24,190 |
| **P5-8** | - | 15458 | 15486 | 29 |  |  |  |
| **gene 5-8** | + | 16128 | 16574 | 447 | hypothetical protein | No homologs | 17,761 |
| **gene 5-7** Exon 1 | + | 16675 | 17565 | 891 | ribonucleotide-diphosphate reductase subunit alpha subunit | nrdA ; Sequence similarity to: YP_001219322.1 ribonucleotide-diphosphate reductase subunit alpha [Candidatus Vesicomyosocius okutanii HA] |  |
| **Intein** | - | 17566 | 18072 | 507 |  |  |  |
| **Exon 2** | + | 18073 | 18495 | 423 | ribonucleotide-diphosphate reductase subunit alpha subunit | ndrA |  |
| **gene 5-6** | + | 19096 | 19746 | 651 | hypothetical protein | No homologs | 25,398 |
| **Exon 3** | + | 19865 | 20413 | 549 | ribonucleotide-diphosphate reductase subunit alpha subunit |  | 85,680 |
| **Intein** | - | 20414 | 20851 | 438 |  |  |  |
| **Exon 4** | + | 20852 | 21247 | 396 | ribonucleotide-diphosphate reductase subunit alpha subunit |  |  |
| **gene 5-5** | + | 21368 | 22045 | 678 | hypothetical protein | No homologs | 26,972 |
| **gene 5-4** | + | 22082 | 22912 | 831 | hypothetical protein | No homologs | 32,171 |
| **gene 5-3** | + | 22967 | 23476 | 510 | NrdG or pyruvate formate-lyase activating enzyme | YP_878525.1 pyruvate formate-lyase activating enzyme [Clostridium novyi NT] | 19,678 |
| **gene 5-2** | + | 23530 | 23937 | 408 | hypothetical protein | No homologs | 15,660 |
| **gene 5-1** | + | 23991 | 24176 | 186 | hypothetical protein | No homologs | 6,950 |
| **gene 5-0** | + | 24259 | 24552 | 294 | putative protein | No homologsor RBS | 11,066 |
|  |  |  |  |  |  |  |  |
| **CONTIG 4** | | | | | | | |
| **ORFs** | **Strand** | **Start** | **End** | **AA Length** | **Product** | **Note (Sequence similarity to):** | **MW** |
|  |  |  |  |  |  |  |  |
| **gene 4-21** | + | 397 | 936 | 540 | hypothetical protein | No homologs | 21,555 |
| **P4-20** | + | 909 | 937 |  |  |  |  |
| **gene 4-20** | + | 971 | 1204 | 234 | hypothetical protein | No homologs | 9,030 |
| **gene 4-19** | + | 1229 | 1522 | 297 | hypothetical protein | No homologs | 10,753 |
| **gene 4-18** | - | 1593 | 2507 | 915 | hypothetical protein | No homologs | 34,571 |
| **gene 4-17** | + | 2630 | 2743 | 114 | hypothetical protein | No homologs | 4,279 |
| **gene 4-16** | + | 2745 | 3209 | 465 | hypothetical protein | No homologs | 18,288 |
| **gene 4-15** | + | 3228 | 3476 | 249 | hypothetical protein | No homologs | 9,628 |
| **gene 4-14** | + | 3491 | 4183 | 693 | RecB family exonuclease | YP_001827363.1 hypothetical protein SGR_5851 [Streptomyces griseus subsp. griseus NBRC 13350] | 27,258 |
| **gene 4-13** | + | 4177 | 5595 | 1419 | DNA helicase UvsW | YP_195146.1 DNA helicase UvsW [Synechococcus phage S-PM2] | 54,226 |
| **P4-12** | + | 5767 | 5795 | 29 |  |  |  |
| **gene 4-12** | + | 5933 | 6361 | 429 | hypothetical protein | No homologs | 16,581 |
| **gene 4-11** | + | 6372 | 7130 | 759 | hypothetical protein | No homologs | 28,400 |
| **gene 4-10** | + | 7254 | 8582 | 1329 | DNA topoisomerase II | NP_899529.1 topoisomerase II small subunit [Vibrio phage KVP40] | 50,973 |
| **gene 4-9** | + | 8595 | 9491 | 897 | putative ATP-dependent Clp protease | YP_006870.1 putative ATP-dependent Clp protease [Enterobacteria phage T5] | 34,235 |
| **gene 4-8** | + | 9565 | 10479 | 915 | hypothetical protein | No homologs | 32,251 |
| **gene 4-7** | + | 10511 | 11362 | 852 | DNA replication origin-binding helicase | NP_045350.1| DNA replication origin-binding helicase [Bovine herpesvirus 1] | 32,787 |
| **gene 4-6** | + | 11355 | 11753 | 399 | hypothetical protein | No homologs | 15,366 |
| **P4-5** | + | 11778 | 11805 | 28 |  |  |  |
| **P4-5'** | + | 11779 | 11805 | 27 |  |  |  |
| **gene 4-5** | + | 11831 | 12256 | 426 | hypothetical protein | No homologs | 16,287 |
| **gene 4-4** | + | 12291 | 12524 | 234 | putative phosphoesterase | YP_002164759.1 possible phosphoesterase [Fusobacterium nucleatum subsp. polymorphum ATCC 10953] | 9,036 |
| **gene 4-3** | + | 12515 | 12829 | 315 | hypothetical protein | No homologs | 12,129 |
| **gene 4-2** | + | 12848 | 13033 | 186 | hypothetical protein | No homologs | 6,950 |
| **gene 4-1** | + | 13058 | 14074 | 1017 | radical SAM domain protein | YP_001617331.1 hypothetical protein sce6682 [Sorangium cellulosum 'So ce 56'] | 39,381 |
| **gene 4-0** | + | 14147 | 14695 | 549 | thymidine kinase | NP_078433.1 thymidine kinase [Ureaplasma parvum serovar 3 str. ATCC 700970] | 21,170 |
|  |  |  |  |  |  |  |  |
|  |  |  |  |  |  |  |  |
| **CONTIG 3** | | | | | | | |
| **ORFs** | **Strand** | **Start** | **End** | **AA Length** | **Product** | **Note (Sequence similarity to):** | **MW** |
| **gene 3-26** | + | 76 | 855 | 780 | hypothetical protein | No homologs | 29,741 |
| **gene 3-25** | + | 1006 | 1914 | 909 | NAD-dependent epimerase dehydratase | YP_001278394.1 NAD-dependent epimerase/dehydratase [Roseiflexus sp. RS-1] | 34,505 |
| **gene 3-24** | + | 1952 | 2524 | 573 | LmbE-like protein | YP_001324623.1 LmbE family protein [Methanococcus aeolicus Nankai-3] | 22,053 |
| **gene 3-23** | + | 2561 | 3106 | 546 | putative polysaccharide deacetylase | YP_359146.1 polysaccharide deacetylase family protein [Carboxydothermus hydrogenoformans Z-2901] | 21,455 |
| **gene 3-22** | + | 3173 | 3811 | 639 | thymidylate synthase | FAD-dependent thymidylate synthase [Campylobacter fetus subsp. fetus 82-40] | 24,421 |
| **gene 3-21** | + | 3889 | 4086 | 198 | hypothetical protein | No homologs | 8,067 |
| **gene 3-20** | + | 4181 | 5290 | 1110 | ribonucleotide-diphosphate reductase subunit beta | YP_717864.1 ribonucleotide reductase subunit B , NrdB [Synechococcus phage syn9] | 43,196 |
| **gene 3-19** | + | 5440 | 5847 | 408 | hypothetical protein | No homologs | 15,719 |
| **gene 3-18** | - | 5910 | 7058 | 1149 | hypothetical protein | No homologs | 44,297 |
| **gene 3-17** | - | 7058 | 7870 | 813 | hypothetical protein | No homologs | 31,530 |
| **gene 3-16** | + | 8070 | 12689 | 4620 | conserved hypothetical protein | YP_179433.1 hypothetical protein CJE1447 [Campylobacter jejuni RM1221] at C-terminus | 170,767 |
| **gene 3-15** | + | 12988 | 14358 | 1371 | tail sheath stabilizer and completion protein | NP_899598.1 tail sheath stabilizer and completion protein gp15 [Vibrio phage KVP40] | 53,154 |
| **gene 3-14** | + | 14367 | 15119 | 753 | tail tube protein | YP_195137.1 tail tube protein gp19 [Synechococcus phage S-PM2] | 27,438 |
| **gene 3-13** | + | 15304 | 15810 | 507 | DNA topoisomerase II ATP-hydrolyzing DNA gyrase subunit B | YP_001812510.1 DNA gyrase, B subunit [Exiguobacterium sibiricum 255-15] | 19,480 |
| **gene 3-12** | + | 15950 | 16876 | 927 | single-stranded DNA binding protein | YP_717676.1 single-stranded DNA binding protein [Synechococcus phage syn9] gp32 . | 34,147 |
| **gene 3-11** | + | 16917 | 17189 | 273 | chaperonin Cpn10 homolog | YP_001636475.1 chaperonin Cpn10 [Chloroflexus aurantiacus J-10-fl] | 10,338 |
| **gene 3-10** | + | 17190 | 17705 | 516 | conserved hypothetical "protein polydeoxyadenosyl region" | NP_734665.1 hypothetical protein gbs0195 [Streptococcus agalactiae NEM316] | 19,451 |
| **miscfeature** | + | 17757 | 17870 | 114 |  | polydeoxyadenosyl region |  |
| **gene 3-9** | + | 17884 | 19488 | 1605 | hypothetical protein | No homologs | 60,318 |
| **gene 3-8** | + | 19660 | 20739 | 1080 | RnlA RNA ligase 1 and tail fiber attachment catalyst | YP_001205566.1 putative RNA ligase [Bradyrhizobium sp. ORS278] | 42,018 |
| **gene 3-7** | + | 20844 | 21296 | 453 | putative xanthine-guanine phosphoribosyltransferase | YP_432394.2 xanthine-guanine phosphoribosyltransferase [Hahella chejuensis] | 17,708 |
| **gene 3-6** | + | 21408 | 21647 | 240 | hypothetical protein | No homologs | 9,591 |
| **gene 3-5** | + | 21803 | 22915 | 1113 | radical SAM domain protein | YP_001255556.1 radical SAM domain protein [Clostridium botulinum A str. ATCC 3502]; no RBS | 43,483 |
| **gene 3-4** | + | 23053 | 23532 | 480 | hypothetical protein | No homologs | 17,337 |
| **gene 3-3** | + | 23564 | 25264 | 1701 | conserved hypothetical protein | YP_214227.1 hypothetical protein PSSP7_050 [Cyanophage P-SSP7] | 60,967 |
| **gene 3-2** | + | 25221 | 26516 | 1296 | hypothetical protein | YP_047607.1 hypothetical protein ACIAD3087 [Acinetobacter sp. ADP1] | 45,642 |
| **gene 3-1** | + | 26544 | 27476 | 933 | baseplate tail tube cap | YP_214242.1 T4-like baseplate tail tube cap gp48 [Prochlorococcus phage P-SSM2] | 34,089 |
| **gene 3-0** | + | 27485 | 27976 | 492 | hypothetical protein | No homologs | 19,413 |
|  |  |  |  |  |  |  |  |
