# Supplementary material for: The genome and proteome of a Campylobacter coli bacteriophage vB_CcoM-IBB_35 reveal unusual features
Source: Virol J. 2012 Jan 27;9:35. doi: 10.1186/1743-422X-9-35 (PMC3322345; doi:10.1186/1743-422X-9-35)
Supplement: Additional file 2 — Table S2 Phage IBB_35 genome annotation. [file 1743-422X-9-35-S2.DOC]

Table S2 – Identification of IBB_35 predicted structural proteins

| **Gene** | **Putative Function** | **MW (KDa)** | **Sequence coverage (%)** |
| --- | --- | --- | --- |
|  |  |  |  |
| **3-16** | conserved hypothetical protein | 170,767 | 9.9 |
| **2-5** | gp6, base plate wedge | 138,284 | 16.9 |
| **2-2** | hypothetical protein | 120,475 | 1.2 |
| **1-57** | conserved hypothetical protein | 92,730 | 12.3 |
| **1-61** | hypothetical protein | 64,565 | 12.5 |
| **1-8** | gp18, tail sheath protein | 64,208 | 11.8 |
| **2-20** | gp20, portal vertex protein of head | 64,126 | 2.5 |
| **3-9** | hypothetical protein | 60,318 | 2.6 |
| **2-15** | gp18, tail sheath protein | 59,193 | 3.5 |
| **1-59** | hypothetical protein | 57,159 | 9.1 |
| **1-7** | gp18, T4-like tail sheath protein | 56,184 | 30.9 |
| **1-6** | gp23, major capsid protein | 48,563 | 48.9 |
| **5-13** | hypothetical protein | 46,414 | 5.5 |
| **2-11** | hypothetical protein | 45,586 | 14.0 |
| **1-56** | hypothetical protein | 44,446 | 17.0 |
| **2-23** | virion structural protein | 44,049 | 13.2 |
| **2-1** | putative protein | 40,671 | 3.5 |
| **2-25** | hypothetical protein | 39,734 | 2.9 |
| **4-18** | hypothetical protein | 34,571 | 17.1 |
| **3-1** | gp48, baseplate tail tube cap | 34,089 | 4.5 |
| **1-63** | hypothetical protein | 28,117 | 5.2 |
| **3-14** | gp19, tail tube protein | 27,438 | 4.8 |
| **1-54** | hypothetical protein | 27,043 | 6.7 |
| **5-24** | gp13, neck protein | 26,874 | 13.2 |
| **2-3** | hypothetical protein | 25,781 | 20.4 |
| **5-10** | hypothetical protein | 25,731 | 19.2 |
| **2-4** | conserved hypothetical protein | 25,471 | 7.7 |
| **2-54** | gp21, T4-like prohead core scaffold and protease | 24,904 | 6.7 |
| **5-30** | putative transcriptional regulator, ExsB homolog | 23,690 | 4.6 |
| **2-38** | minor tail protein | 23,617 | 38.1 |
| **5-25** | hypothetical protein | 23,245 | 25.1 |
| **2-26** | hypothetical protein | 21,911 | 19.4 |
| **1-14** | gp19, tail tube protein | 21,708 | 12.8 |
| **1-30** | conserved hypothetical protein | 21,402 | 7.2 |
| **5-12** | hypothetical protein | 20,056 | 8.2 |
| **3-0** | hypothetical protein | 19,413 | 7.4 |
| **2-24** | hypothetical protein | 10,994 | 23.7 |
| **5-15** | putative protein | 10,984 | 26.8 |
|  |  |  |  |
